# *Bradyrhizobium ganzhouense* sp. nov., an effective symbiotic bacterium isolated from *Acacia melanoxylon* R. Br. nodules

**DOI:** 10.1099/ijs.0.056564-0

**Published:** 2014-06

**Authors:** Jun Kun Lu, Ya Jing Dou, Ya Jie Zhu, Sheng Kun Wang, Xin Hua Sui, Li Hua Kang

**Affiliations:** 1 Research Institute of Tropical Forestry, Guangzhou 510520, PR China; 2 State Key Laboratories for Agrobiotechnology, College of Biological Sciences, China Agricultural University, Beijing 100193, PR China

## Abstract

Three slow-growing rhizobial strains, designated RITF806^T^, RITF807 and RITF211, isolated from root nodules of *Acacia melanoxylon* grown in Ganzhou city, Jiangxi Province, China, had been previously defined, based on amplified 16S rRNA gene restriction analysis, as a novel group within the genus *Bradyrhizobium*. To clarify their taxonomic position, these strains were further analysed and compared with reference strains of related bacteria using a polyphasic approach. According to 16S rRNA gene sequence analysis, the isolates formed a group that was closely related to ‘*Bradyrhizobium rifense*’ CTAW71, with a similarity value of 99.9 %. In phylogenetic analyses of the housekeeping and symbiotic gene sequences, the three strains formed a distinct lineage within the genus *Bradyrhizobium*, which was consistent with the results of DNA–DNA hybridization. In analyses of cellular fatty acids and phenotypic features, some differences were found between the novel group and related species of the genus *Bradyrhizobium*, indicating that these three strains constituted a novel group distinct from any recognized species of the genus *Bradyrhizobium*. Based on the data obtained in this study, we conclude that our strains represent a novel species of the genus *Bradyrhizobium*, for which the name *Bradyrhizobium ganzhouense* sp. nov. is proposed, with RITF806^T^ ( = CCBAU 101088^T^ = JCM 19881^T^) as the type strain. The DNA G+C content of strain RITF806^T^ is 64.6 mol% (*T*_m_).

Australian blackwood (*Acacia melanoxylon* R. Br.) has its origin in the temperate forests of eastern Australia but it is a versatile and highly adaptive tree species that occurs naturally across a wide range of Australian forest ecosystems (Searle, 2000). Blackwood is also grown in plantations, including as an exotic in several countries, in particular because of its ornamental value and the quality of its dark wood (see Bradbury *et al.* 2010). Like most species of the genus *Acacia*, blackwood forms nodules in symbiosis with rhizobia (Dou *et al.*, 2012). As a result of this nitrogen-fixing symbiosis, it plays an important role in natural ecosystems by improving soil fertility. In China, blackwood was introduced as a premium-grade furniture timber at the end of the nineteenth century. At present in China, blackwood is found in pure stands in Jiangxi, Fujian, Guangdong, Guangxi and Hainan Provinces.

The rhizobia associated with *Acacia melanoxylon* collected from soils of seedling nurseries or plantations have not, to our knowledge, previously been studied and no molecular evolutionary characterization of these bacteria has been reported. In China, *Acacia melanoxylon* forms nodules even when not inoculated, but limited information is available about the rhizobia which form these symbioses. In an earlier study, 174 isolates originating from *Acacia melanoxylon* growing in China were found to cluster into three genotypic groups according to 16S rRNA analysis; each group included isolates from different sites (Dou *et al.*, 2012). Our objective in this study was to characterize isolates from nodules of plants growing in Chinese soils using a polyphasic approach. This study demonstrated that these three strains represent a novel species phylogenetically, which belongs to the genus *Bradyrhizobium*. We propose the name *Bradyrhizobium ganzhouense* sp. nov. for this species.

The three test strains and reference strains were obtained from the Research Institute of Tropical Forestry and the State Key Laboratory of Agrobiotechnology. They were maintained on YMA medium (Vincent, 1970) at 4 °C during temporary storage. Genomic DNA was extracted from the strains according to the protocol of Chen & Kuo (1993) and was used as a template for the amplification of different genes and specific DNA fragments. For phylogenetic analyses, the following targets were amplified from the test strains: (i) the 16S rRNA gene (~1450 nt) with primers 27F and 1492R (DeLong, 1992); (ii) partial sequences of housekeeping genes *recA* (~600 nt), *glnII* (~680 nt) and *atpD* (~530 nt), with the amplifying primers and conditions described by Vinuesa *et al.* (2005); and (iii) *nodC* (~900 bp) and *nifH* (~800 bp) using primer pairs nodCF540/nodCR1160 and nifHF/nifHR and the protocol of Laguerre *et al.* (2001).

All PCR products were sequenced with a BigDye terminator v3.1 kit using an ABI-PRISM 3730 Genetic Analyzer (ABI) with protocols recommended by the manufacturer. Gene sequences of type strains were obtained from the GenBank database. Neighbour joining (NJ) and maximum-likelihood (ML) phylogenies were inferred with mega 5.1.

The results of phylogenetic analysis of the 16S rRNA gene (~1250 nt) indicated that strains RITF806^T^, RITF807 and RITF211 represented a member of the genus *Bradyrhizobium* and had 99.9 % sequence similarity to ‘*Bradyrhizobium rifense*’ CTAW71^T^ (Table 1, Fig. 1).

**Table 1.** Range of percentage nucleotide identity within *Bradyrhizobium ganzhouense* sp. nov. and between *B. ganzhouense* strains and the type strains of other species of the genus *Bradyrhizobium* in the 16S rRNA gene and three protein-coding genes

Species	Gene*			
	16S rRNA	*recA*	*glnII*	*atpD*
Within *B. ganzhouense*†	99.9–100	99.7–100	98.6–100	97.4–97.9
Between *B. ganzhouense* and:				
‘*B. arachidis*’	99.4–99.5	92.7–93.0	93.3–93.5	95.5–96.0
*B. betae*	99.5–99.6	92.2–92.7	94.2–95.2	96.0–96.3
*B. canariense*	99.0	92.1–92.4	95.2–95.6	93.1–94.4
*B. cytisi*	99.5–99.6	91.8–92.1	95.4–96.2	92.9–94.2
*B. diazoefficiens*	99.6–99.7	92.7–93.0	96.0–97.0	96.0–96.8
*B. elkanii*	96.9–97.0	89.9–90.1	88.1–88.9	92.1–92.6
*B. huanghuaihaiense*	99.3–99.4	90.7–91.0	96.0–96.4	95.2–96.3
*B. japonicum*	99.2–99.3	91.8–92.1	96.0–97.0	93.1–93.7
*B. jicamae*	97.0–97.1	87.6–87.9	86.9–87.7	92.6–93.4
*B. lablabi*	97.1–97.2	89.9–90.1	87.9–88.9	91.5–92.6
*B. liaoningense*	99.3–99.4	91.8–92.1	94.4–95.0	93.7–94.4
*B. oligotrophicum*	98.7–98.8	87.6–87.9	84.5–85.5	91.0–91.8
*B. pachyrhizi*	97.0–97.1	89.0–89.3	87.7–88.5	92.3–92.9
‘*B. rifense*’	99.9	92.1–92.4	97.4–97.6	96.0–96.8
*B. yuanmingense*	99.1–99.2	91.3–91.5	94.4–94.6	92.6–93.7
*E. fredii*	89.1	78.6–78.9	85.9–86.1	77.5–79.1

* Length of the aligned regions (bp): 16S rRNA gene (1250), *recA* (355), *glnII* (497) and *atpD* (391).

† Three strains: RITF806^T^, RITF807 and RITF211.

**Fig. 1.**
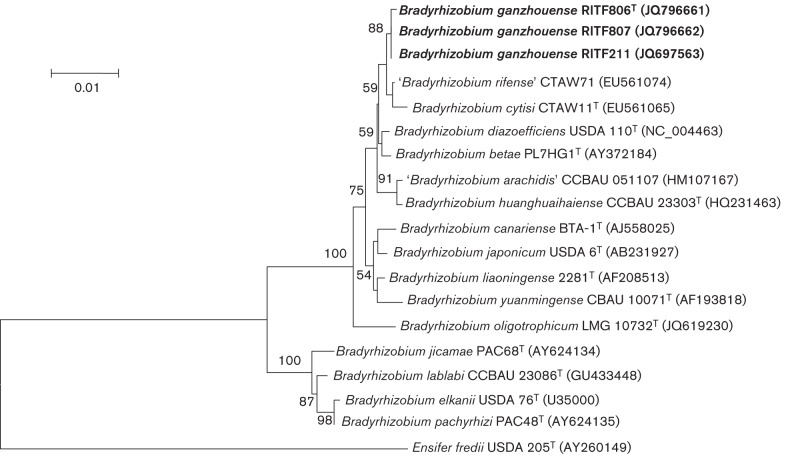
16S rRNA gene neighbour-joining phylogenetic tree (1250 nt) showing the relationships between test strains of *Bradyrhizobium ganzhouense* sp. nov. and other species of the genus *Bradyrhizobium*. *Ensifer fredii* USDA 205^T^ was used as an outgroup. The tree was derived from a distance matrix (Kimura’s two-parameter model). Bootstrap support values higher than 50 % (calculated for 1000 subsets) are indicated at nodes. Bar, 0.01 expected changes per site.

The three strains were found to have nearly identical sequences for three housekeeping genes (*recA*, *glnII and atpD*). For each of the three genes, the phylogenetic relationships were consistent between the NJ and ML trees (data not shown). In the multilocus sequence analysis (MLSA), the ML tree reconstructed on the basis of the combined sequences of the three housekeeping genes (Fig. 2) had the same topology as the NJ tree (data not shown). The strains of the novel group in the concatenated tree formed a distinct lineage from other previously defined species (Fig. 2). The sequence similarities of the *recA*, *glnII* and *atpD* genes were 97.4–100 % among the three novel strains and were <97.6 % between the novel group and reference strains of related species (Table 1). The most similar strains to RITF806^T^ were *Bradyrhizobium diazoefficiens* USDA 110^T^ for the *recA* gene (93.0 % similarity), and ‘*B. rifense*’ CTAW71^T^ for the *glnII* gene (97.6 % similarity) and for the *atpD* gene (96.2 % similarity) (Table 1). The novel strain RITF806^T^ was most similar to ‘*B. rifense*’ CTAW71^T^ and *B. diazoefficiens* USDA 110^T^ according to the MLSA (95.8 % similarity).

**Fig. 2.**
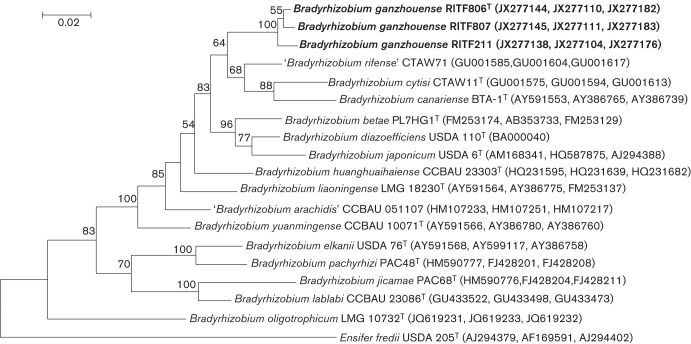
Maximum-likelihood tree based on partial concatenated sequences of *recA* (355 nt), *glnII* (497 nt) and *atpD* (391 nt) genes of *Bradyrhizobium ganzhouense* sp. nov. and closely related species within the genus *Bradyrhizobium*. *Ensifer fredii* USDA 205^T^ was used as an outgroup. Bootstrap support values higher than 50 % (calculated for 100 subsets) are indicated at nodes. Bar, 2 nt substitutions per 100 nt.

Symbiotic genes cannot be used as gene markers for taxonomic differentiation because they are normally located in transferable elements (plasmids or symbiotic islands). However, they can provide valuable information about rhizobial species (Laguerre *et al.*, 2001). In the phylogenetic analysis of both *nodC* and *nifH* genes, the novel strains examined in the present study always formed a distinctive lineage that differed from all defined species. In the *nodC* phylogenetic tree (Fig. S1 available with the online Supplementary Material), the three test strains had identical sequences, which were most similar to that of *Mesorhizobium* sp. CCBAU 33477 (79.5 % similarity), which had been isolated from effective nodules of *Astragalus sinicus* in Southern China (data from GenBank). For the *nifH* gene, the highest similarity (around 90 %) was to *Bradyrhizobium lablabi* CCBAU 23086^T^ (Fig. S2), isolated from root nodules of *Lablab purpureus* and *Arachis hypogaea* grown in the Anhui and Sichuan provinces of China (Chang *et al.*, 2011).

DNA–DNA hybridization is an important index for the definition of bacterial species (Wayne *et al.*, 1987). In the present study, total DNA was extracted from the three test strains and reference strains using the method of Marmur (1961). DNA–DNA relatedness between RITF806^T^ and other strains was estimated using renaturation-rate technology (DeLey *et al.*, 1970). The DNA–DNA relatedness of RITF806^T^ to RITF807 and RITF211 was 85.23 and 78.79 %, respectively, indicating that both strains belong to the same species. The DNA–DNA relatedness between RITF806^T^ and reference strains *Bradyrhizobium cytisi* CTAW11^T^, *B. huanghuaihaiense* CCBAU 23303^T^, *B. diazoefficiens* USDA 110^T^ and ‘*B. rifense*’ CTAW71^T^ were 58.11, 54.61, 53.45 and 51.89 %, respectively (Table 2), which are much lower than the threshold of 70 % recommended for species definition (Wayne *et al.*, 1987).

**Table 2.** DNA–DNA hybridization values within the novel species *Bradyrhizobium ganzhouense* sp. nov. and between these taxa and phylogenetically related species of the genus *Bradyrhizobium* DNA G+C content data for *B. cytisi* was taken from Chahboune *et al.* (2011), data for *B. huanghuaihaiense* from Zhang *et al.* (2012), data for *B. diazoefficiens* from Delamuta *et al.* (2013), data for ‘*B. rifense’* from Chahboune *et al.* (2012), *B. canariense* from Vinuesa *et al.* (2005), *B. japonicum* from Yao *et al.* (2002) and *B. betae* from Rivas *et al.* (2004).

Strain providing fixed DNA	DNA–DNA relatedness (%) with RITF806^T^	DNA G+C content* (mol%)
RITF806^T^	100	64.6
RITF807	85.23	65.2
RITF211	78.79	62.8
*B. cytisi* CTAW11^T^	58.11	65.1
*B. huanghuaihaiense* CCBAU 23303^T^	54.61	61.5
*B. diazoefficiens* USDA 110^T^	53.45	63.9
‘*B. rifense*’ CTAW71	51.89	62.7
*B. canariense* LMG 22265^T^	38.95	63.8
*B. japonicum* USDA 6^T^	35.13	63.3
*B. betae* PL7HG1^T^	34.19	63.7

The G+C content of DNA was determined by the thermal denaturation method (Mandel & Marmur, 1968). The values for strains RITF806^T^, RITF807 and RITF211 were 64.6, 65.2 and 62.8 mol% (*T*_m_), respectively, which is within the range for members of the genus *Bradyrhizobium*.

Cellular fatty acids of strain RITF806^T^ were assayed together with those of *B. cytisi* CTAW11^T^, *B. diazoefficiens* USDA 110^T^ and ‘*B. rifense*’ CTAW71 in order to examine differences between the novel strain and closely related species. The strains were cultured aerobically on YMA medium at 28 °C and cells were collected during the late-exponential phase of growth. Fatty acid methyl esters were prepared and separated using the method described by Sasser (1990) and identified with a MIDI Sherlock Microbial Identification System (Sherlock license CD version 6.0), using the TSBA6 database. A total of nine fatty acids or summed features were detected in strain RITF806^T^ (Table 3). All tested strains contained the fatty acids anteiso-C_14 : 0_, anteiso-C_17 : 0_, C_16 : 0_ and summed feature 8 (C_18 : 1_ω6*c*/C_18 : 1_ω7*c*), but the percentages of these fatty acids varied (Table 3). Summed feature 8 and C_16 : 0_ were the two most abundant fatty acids in all tested strains, except for the *B. cytisi* CTAW11^T^, as reported previously for members of the genus *Bradyrhizobium* (Tighe *et al.*, 2000).

**Table 3.** Fatty acid profiles of *Bradyrhizobium ganzhouense* sp. nov. RITF806^T^ and related strains of members of the genus *Bradyrhizobium* Strains: 1, *B. ganzhouense* RITF806^T^; 2, *B. cytisi* CTAW11^T^; 3, *B. diazoefficiens* USDA 110^T^; 4, ‘*B. rifense*’ CTAW71; 5, *B. huanghuaihaiense* CCBAU 23303^T^. −, not detected. Data were determined with the MIDI system using the TSBA6 database.

Fatty acid	1	2	3	4	5*
anteiso-C_11 : 0_	−	−	0.66	−	−
anteiso-C_12 : 0_	−	0.33	1.56	−	−
anteiso-C_13 : 0_	−	0.30	2.24	−	−
C_14 : 0_	−	0.16	−	−	0.49
anteiso-C_14 : 0_	1.60	0.3	2.25	1.19	0.14
anteiso-C_15 : 0_	−	0.28	−	1.08	0.27
C_16 : 0_	14.12	9.49	11.58	11.53	10.05
anteiso-C_16 : 0_	0.79	−	0.94	0.50	−
C_16 : 1_ω5*c*	2.99	10.62	−	5.76	3.29
C_16 : 1_ω11*c*	−	0.50	−	−	−
anteiso-C_17 : 0_	0.93	0.17	1.17	0.67	0.12
C_17 : 0_cyclo	−	0.38	−	−	−
C_17 : 1_ω6*c*	−	−	−	−	1.02
C_17 : 1_ω8*c*	−	−	−	−	1.45
C_18 : 0_	−	0.37	1.45	−	0.85
C_18 : 1_ω7*c* 11-methyl	4.59	3.33	−	0.76	−
C_19 : 0_cyclo ω8*c*	3.62	7.07	−	3.19	0.66
Summed features†					
3	1.50	1.38	−	2.24	1.17
8	68.49	65.33	76.43	73.08	75.92

* Data for *B. huanghuaihaiense* was taken from Zhang *et al.* (2012).

† Summed feature 3 comprised C_16 : 1_ω6*c*/C_16 : 1_ω7*c*; summed feature 8 comprised C_18 : 1_ω6*c*/C_18 : 1_ω7*c*.

The polar lipid profiles of cells of strain RITF806^T^ were determined following the protocol described by Minnikin *et al.* (1984) and Zhang *et al.* (2012). The phospholipid profile is shown in Fig. S3. Strain RITF806^T^ contained aminolipid, diphosphatidylglycerol, phosphatidylcholine, phosphatidylethanolamine, phosphatidylglycerol and an unknown polar lipid with phosphatidylcholine and phosphatidylethanolamine as the major components (each representing about 40 % of the total phospholipids).

Phenotypic characterization of the three test strains in this study was based on the API 20NE kit (bioMérieux), according to the manufacturer’s instructions, using YM-minus-mannitol as the basal medium. Carbon-source utilization was determined using a Biolog GN2 microplate (Gram-negative bacterial identification test panel), according to the manufacturer’s instructions. Tolerance to dyes, antibiotics and sodium chloride, and other characteristics were assessed as described by Gao *et al.* (1994). The combination of phenotypic features listed in Table 4 could be used to differentiate the novel strains from species of the genus *Bradyrhizobium* with validly published names.

**Table 4.** Differential characteristics of *Bradyrhizobium ganzhouense* sp. nov. and closely related species Species: 1, RITF806^T^; 2, RITF807; 3, RITF211; 4, *B. cytisi* CTAW11^T^ (Data from Chahboune *et al*., 2011); 5, *B. diazoefficiens* USDA 110^T^ (Delamuta *et al*., 2013); 6, ‘*B. rifense*’ CTAW71 (Chahboune *et al.*, 2012) 7, *B. canariense* LMG 22265^T^ (Vinuesa *et al*., 2005); 8, *B. huanghuaihaiense* CCBAU 23303^T^ (Zhang *et al*., 2012). +, Growth; −, no growth; w, weakly positive; ±, variable.

Characteristic	1	2	3	4	5	6	7	8
Assimilation (as carbon source) of:								
d-Fructose	+	+	+	+	+	+	−	+
d-Glucose	−	−	−	+	w	w	w	w
l-Rhamnose	−	−	−	−	w	w	+	w
*N*-Acetylglucosamine	−	−	−	+	−	+	+	−
Growth on YMA with/at:								
pH 4.5	−	−	−	−	+	+	+	−
1 % NaCl	+	+	+	−	−	−	−	−
37 °C	+	+	+	−	−	−	−	+
100 µg Erythromycin ml^−1^	+	+	+	+	−	+	±	+

The newly isolated strains can be differentiated genotypically and phenotypically from previously described species and we therefore propose naming the new group *Bradyrhizobium ganzhouense* sp. nov.

## Description of *Bradyrhizobium ganzhouense* sp. nov.

*Bradyrhizobium ganzhouense* (gan.zhou.en′se. N.L. neut. adj. *ganzhouense* of or belonging to Ganzhou City, Jiangxi Province, China).

Cells are Gram-negative, aerobic, non-spore-forming rods, 1.29–3.32 µm long and 0.50–0.60 µm wide. Colonies are circular, convex and translucent, 1–2 mm in diameter within 7 days of growth at 28 °C on YMA medium. Grows at pH 5–12, with optimum growth at pH 7.0. Growth occurs at 4 °C, 10 °C, 28 °C and 37 °C (optimally at 28 °C). Grows on YMA in the presence of 3 % NaCl. Cannot tolerate 60 °C for 10 min on YMA. No growth in Luria–Bertani broth. Positive for catalase, oxidase and urease production. Nitrate reduction, Nile blue reduction and methylthionine chloride reduction are negative. Uses Tween 80, d-fructose, α-d-glucose, d-mannitol, d-mannose, citric acid, methyl pyruvate, d-galactonic acid lactone, d-galacturonic acid, d-gluconic acid, dl-lactic acid, d-saccharic acid, succinic acid, d-alanine and glycerol as carbon sources. Does not grow on d-glucose, maltose, l-rhamnose, *N*-acetyl-d-galactosamine, *N*-acetyl-d-glucosamine, i-erythritol, *myo*-inositol, α-lactose, lactulose, melibiose, raffinose, sucrose, xylitol, inosine, uridine or thymidine. Resistant to the following antibiotics (μg ml^−1^) erythromycin (100), kanamycin (100), neomycin sulfate (50), streptomycin (5), chloramphenicol (300), gentamicin (5) and totomycin (100). Summed feature 8 (C_18 : 1_ω6*c*/C_18 : 1_ω7*c*) and C_16 : 0_ are the dominant fatty acids. The polar lipid profile contains aminolipid, diphosphatidylglycerol, phosphatidylcholine, phosphatidylethanolamine and phosphatidylglycerol. Can form effective nodules on its original host plant *Acacia melanoxylon* and other species including *Acacia aneura*, *Acacia victoriae* and *Acacia implexa*, but not *Medicago sativa*, *Pisum sativum*, *Trifolium albus* or *Vigna unguiculata*.

The type strain, RITF806^T^ ( = CCBAU 101088^T^ = JCM 19881^T^), was isolated from effective nodules of *Acacia melanoxylon* R. Br. Its DNA G+C content is 64.6 mol% (*T*_m_).
